# Smad7 and protein phosphatase 1α are critical determinants in the duration of TGF-β/ALK1 signaling in endothelial cells

**DOI:** 10.1186/1471-2121-7-16

**Published:** 2006-03-29

**Authors:** Gudrun Valdimarsdottir, Marie-José Goumans, Fumiko Itoh, Susumu Itoh, Carl-Henrik Heldin, Peter ten Dijke

**Affiliations:** 1 Dept. of Biochemistry and Molecular Biology, Faculty of Medicine, University of Iceland, Vatnsmyrarvegur 16, 101 Reykjavik, Iceland; 2 Heart Lung Center, Department of Cardiology, University Medical Center Utrecht, Heidelberglaan 100, 3584 CX, Utrecht, The Netherlands; 3 Graduate School of Comprehensive Human Sciences, University of Tsukuba, 1-1-1, Tennodai, Tsukuba, Ibaraki 305–8575, Japan; 4 ^4^Ludwig Institute for Cancer Research, Uppsala University, Biomedical Center, S-751 24, Uppsala, Sweden; 5 Molecular Cell Biology, Leiden University Medical Center, Postbus 9600, 2300 RC Leiden, The Netherlands

## Abstract

**Background:**

In endothelial cells (EC), transforming growth factor-β (TGF-β) can bind to and transduce signals through ALK1 and ALK5. The TGF-β/ALK5 and TGF-β/ALK1 pathways have opposite effects on EC behaviour. Besides differential receptor binding, the duration of TGF-β signaling is an important specificity determinant for signaling responses. TGF-β/ALK1-induced Smad1/5 phosphorylation in ECs occurs transiently.

**Results:**

The temporal activation of TGF-β-induced Smad1/5 phosphorylation in ECs was found to be affected by *de novo *protein synthesis, and ALK1 and Smad5 expression levels determined signal strength of TGF-β/ALK1 signaling pathway. Smad7 and protein phosphatase 1α (PP1α) mRNA expression levels were found to be specifically upregulated by TGF-β/ALK1. Ectopic expression of Smad7 or PP1α potently inhibited TGF-β/ALK1-induced Smad1/5 phosphorylation in ECs. Conversely, siRNA-mediated knockdown of Smad7 or PP1α enhanced TGF-β/ALK1-induced signaling responses. PP1α interacted with ALK1 and this association was further potentiated by Smad7. Dephosphorylation of the ALK1, immunoprecipitated from cell lysates, was attenuated by a specific PP1 inhibitor.

**Conclusion:**

Our results suggest that upon its induction by the TGF-β/ALK1 pathway, Smad7 may recruit PP1α to ALK1, and thereby control TGF-β/ALK1-induced Smad1/5 phosphorylation.

## Background

Transforming growth factor-β (TGF-β) elicits its cellular effects through activation of type I and type II serine/threonine kinase receptors [1,2]. The constitutively active type II receptor phosphorylates specific serine and threonine residues in the juxtamembrane region (so-called GS domain) of the type I receptor. Type I receptor acts downstream of type II receptor (Tβ R-II) and has been shown to determine signaling specificity within the heteromeric receptor complex. In most cell types, TGF-β signals via TGF-β type I receptor (Tβ R-I), also termed activin receptor-like kinase 5 (ALK5). In endothelial cells (ECs), however, TGF-β can signal via Tβ R-II and two different type I receptors, i.e. the broadly expressed ALK5 and the EC-restricted ALK1. Whereas ALK5 inhibits EC migration and proliferation, ALK1 stimulates both these processes [3]. The activated type I receptor propagates the signal through phosphorylation of specific receptor-regulated Smads (R-Smads). Whereas ALK5 induces the phosphorylation of Smad2 and Smad3, ALK1 mediates the activation of Smad1 and Smad5 [4,5]. Activated R-Smads can assemble into heteromeric complexes with common partner (Co-) Smad, *i.e*. Smad4 and translocate into the nucleus where they regulate the transcription of target genes [1,2].

I-Smads (Smad6 and Smad7) are natural inhibitors of TGF-β signaling that prevent the activation of R- and Co-Smads [6-8]. They do so by interacting efficiently with the activated type I receptors preventing access and phosphorylation of R-Smads by the activated type I receptors. Smad6 has also been found to exert its inhibitory effect on signaling by competing with Smad4 for heteromeric complex formation with activated Smad1 [9] and by recruiting the co-repressor CtBP and thereby repress transcription [10,11]. I-Smads were found to interact with Smad ubiquitination-related factors, Smurfs, which are HECT-domain ubiquitin ligases that target the TGF-β receptors for degradation [12,13]. The expression of I-Smads is quickly induced upon stimulation by members of the TGF-β family and upon shear stress of the endothelium [14,15]. Thus, I-Smads may be part of negative feedback control mechanisms.

A key event in TGF-β signaling is serine phosphorylation of Tβ R-I by Tβ R-II, and of R-Smads by Tβ R-I. These phosphorylations are tightly controlled, *e.g*. the immunophilin FKBP-12 binds to Tβ R-I and thereby inhibits phosphorylation of Tβ R-I by Tβ R-II [16]. C-terminal phosphorylation of Smad2 and Smad3 is strongly facilitated by Smad anchor for receptor activation (SARA)[17].

Serine/threonine protein phosphatases (PPs) are likely involved in the dephosphorylation of these phosphorylated signaling components. PPs consist of a catalytic subunit that binds to one or two regulatory subunits that generate holoenzymes with unique localizations and specificities [18]. One of the major PPs is PP1 consisting of a PP1 catalytic subunit (PP1c) that can form complexes with more than 50 regulatory subunits [19]. Four mammalian isoforms of the PP1c gene have thus far been described, *i.e*. PP1α, PP1β and two splice variants of PP1γ. Studies in *Drosophila melanogaster *suggest that PP1 binds to the decapentaplegic (dpp) type I receptor with the aid of SARA and negatively regulates dpp signaling [20]. Very recently, it was reported that the TGF-β- induced Smad7 can interact with the growth arrest and DNA damage protein 34 (GADD34) (21), which is a regulatory subunit of PP1. The Smad7- GADD34 complex was shown to recruit PP1c to Tβ R-I, and thereby dephosphorylate and inactivate it [22].

In the present report, we have investigated the molecular mechanisms that underlie the TGF-β-induced transient ALK1-mediated Smad1/5 phosphorylation versus sustained ALK5-mediated Smad2 phosphorylation in ECs. Analysis of the effect of various chemical inhibitors on the TGF-β/ALK1 response, suggested an important contribution of PP1 in the negative regulation of ALK1 signaling, but not ALK5 signaling in ECs. Our data suggest that Smad7, induced by ALK1 activation, recruits PP1α to ALK1 and thereby inhibits TGF-β/ALK1-induced Smad1/5 phosphorylation in ECs.

## Results

### Negative regulation of TGF-β-induced Smad1/5 phosphorylation is dependent on protein synthesis in ECs

**Figure 1:**
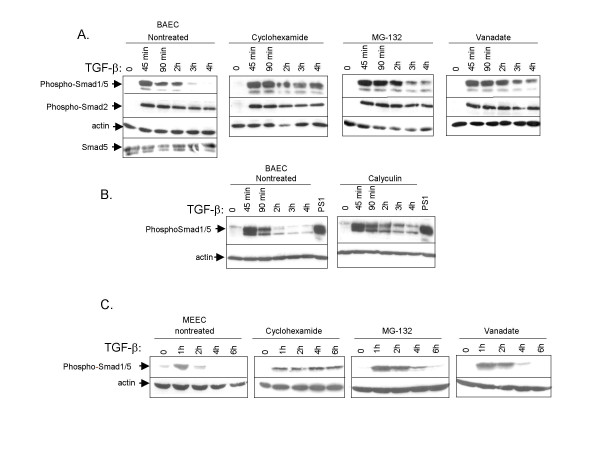
**Negative regulation of TGF-β-induced Smad1/5 phosphorylation.**(A) *First panel*. BAECs were stimulated with TGF-β (1 ng/ml) for different time periods at 37°C before lysis. Whole cell lysate was sonicated and fractionated by SDS-PAGE and blotted. The filters were incubated with PS1 antibody that specifically recognizes phosphorylated Smad1/5, PS2 antibody, which specifically recognizes phosphorylated Smad2, and antisera against Smad5. The blots were incubated with an actin antibody as a control for protein loading. *Second panel*. BAECs were treated with the protein synthesis inhibitor cyclohexamide (10 ng/ml) 30 minutes before they were stimulated with TGF-β. The filters were incubated with PS1, PS2 or actin antibodies. *Third panel*. BAECs were treated with the proteasome inhibitor, MG-132 (10 nM), for 30 min before they were stimulated with TGF-β. The filters were incubated with PS1, PS2 or actin antibodies. *Last panel*. BAECs were pre-treated with a phosphatase inhibitor, sodium orthovanadate (1 mM) 30 min prior to stimulation with TGF-β following the same procedure as in Fig. 1A. The filters were incubated with PS1, PS2 or actin antibody. (B) BAECs were pre-treated (right panel) or not (left panel) with the Ser/Thr phosphatase inhibitor calyculin (1 nM) 30 min prior to stimulation with TGF-β for different time periods before lysis. The filters were incubated with PS1 or actin antibody. (C) MEECs were pre-treated with cyclohexamide, MG-132, vanadate or not, and then stimulated with TGF-β for different time periods as in Fig. 1A.

Recently we showed that upon TGF-β stimulation in primary ECs, Smad2 phosphorylation is stable for at least 6 hours whereas Smad1/5 phosphorylation is transient and absent 3 h after stimulation [3]. In order to find out if this short duration of TGF-β-induced Smad1/5 activation is dependent on induction of newly synthesized proteins, we treated bovine aortic endothelial cells (BAECs) with the protein synthesis inhibitor, cyclohexamide, and examined the kinetics of Smad phosphorylation after TGF-β stimulation. In the non-treated cells, Smad1/5 phosphorylation reached peak levels after 45 min of TGF-β stimulation and declined thereafter until no phosphorylation was detected after 3 h (Fig. 1A, first panel). Compared to control cells, the TGF-β-induced Smad1/5 phosphorylation in cyclohexamide-treated cells was more sustained and lasted at least 4 h (Fig. 1A, second panel). Total Smad levels were not found to be significantly affected upon cycloheximide treatment during the course of the experiment. Similar results were obtained in another type of ECs, the mouse embryonic ECs (MEECs) (Fig. 1C). These results suggest that the TGF-β induced Smad1/5 signaling is inhibited by newly synthesized inhibitory proteins.

### Signal strength of TGF-β/ALK1 pathway in ECs is critically dependent on ALK1 and Smad5 expression levels

**Figure 2:**
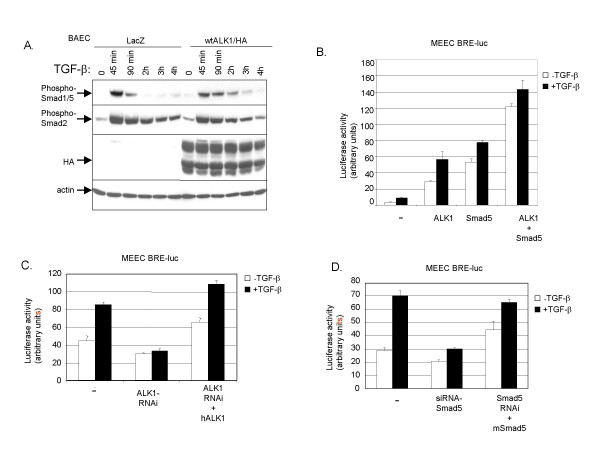
**ALK1 and Smad are critically important in Smad1/5 phosphorylation in ECs. **(*A*) BAECs were adenovirally infected with wild-type ALK1/HA or LacZ construct at MOI of 1000. Fresh medium containing 10% FBS was added 16 h after infection. Eight hours later, the cells were starved overnight and then stimulated with TGF-β (1 ng/ml) for the indicated time periods and TGF-β-induced Smad1/5 phosphorylation was measured. Cells were lysed, sonicated and fractionated by SDS-PAGE. The gels were then subjected to immunoblotting. The filters were incubated with PS1, PS2, HA or actin antibody. (*B*) MEECs were transfected with BRE-luc in the absence or presence of ALK1 or Smad5, or both. After 48 h, cells were extensively washed. Then, cells were stimulated for 8 h with TGF-β, or not, and luciferase activity was measured. Values are corrected for transfection efficiency as measured by β-galactosidase activity. A representative experiment using triplicate samples is shown. (*C*) MEECs were transfected with (BRE)-luc in the absence or presence of ALK1-RNAi. After 48 h, cells were extensively washed. Then, cells were stimulated for 8 h with TGF-β, or not, and luciferase activity was measured. The luciferase activity upon RNAi-mediated knockdown of endogenous mouse ALK1 was rescued by co-transfecting a human ALK1 expression plasmid. Values are corrected for transfection efficiency as measured by β-galactosidase activity. A representative experiment using triplicate samples is shown. (*D*) MEECs were transfected with BRE-luc in the absence or presence of Smad5-RNAi. After 48 h, cells were extensively washed. Then, cells were stimulated for 16 h with TGF-β, or not, and luciferase activity was measured. The luciferase activity upon RNAi-mediated knockdown of endogenous mouse Smad5 was rescued by co-transfecting a mouse Smad5 plasmid. Values are corrected for transfection efficiency as measured by β-galactosidase activity. A representative experiment using triplicate samples is shown.

Previously, we showed that TGF-β-induced Smad1/5 phosphorylation is dependent on ALK1. Treatment of ECs with ALK1 antisense oligonucleotides specifically inhibited TGF-β-induced Smad1/5 phosphorylation [3]. To examine whether intensity and/or duration of signaling is affected by ALK1 and/or Smad5 levels, we initially infected BAECs with adenoviral constructs of wtALK1 or LacZ and analysed TGF-β/Smad phosphorylation. As shown in Figure 2A, whereas Smad1/5 phosporylation had returned to background levels after 90 min of TGF-β stimulation in LacZ infected cells, in cells infected with wild-type (wt) ALK1 adenoviral construct TGF-β-induced Smad1/5 phosphorylation lasted until 3 h.

We strengthened these data by making use of the BRE-luc reporter, which can be used as a transcriptional read-out for TGF-β/ALK1 signaling [3]. TGF-β induction of the BRE-luc reporter was potentiated upon ALK1 and/or Smad5 co-transfection (Fig. 2B) and attenuated upon siRNA-mediated knockdown of ALK1 (Fig. 2C) or Smad5 (Fig. 2D). Inhibition of TGF-β-induced activation of BRE-luc reporter by siRNA-ALK1 could be rescued by cotransfection of human ALK1 or Smad5, respectively (Fig. 2C, D). Taken together, these results demonstrate that TGF-β/ALK1-induced Smad1/5 activation is critically dependent on the ALK1 and Smad5 expression level in ECs.

### Smad7 is induced upon TGF-β/ALK1 signaling in ECs

**Figure 3:**
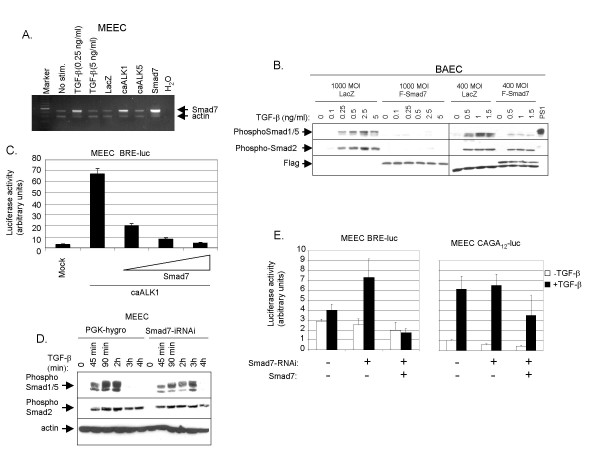
**Smad7 is a potent inhibitor of ALK1-induced Smad1/5 phosphorylation in Ecs.**(*A*) MEECs were stimulated with TGF-β or adenovirally infected at MOI of 500 with LacZ, caALK1, caALK5 and Smad7 (included as a positive control). Forty hours later, the noninfected cells were starved overnight and then stimulated with TGF-β, or not, for 90 min. Cells were lysed, and RNA was isolated and cDNA prepared. Expression of Smad7 was analysed by semi-quantitative RT-PCR. β-actin expression was measured to control for equal loading. The PCR products were loaded on 1% agarose gel and stained with ethidium bromide. (*B*) *Left panel*. BAECs were infected with adenoviral constructs of Flag-Smad7 or lacZ as a control with MOI of 1000. Forty hours later, BAECs were stimulated with a different dose of TGF-β at 37°C before lysis. Whole cell lysate was sonicated and fractionated by SDS-PAGE and blotted. The filters were incubated with PS1, PS2 or α-Flag antibody. *Right panel*. Differential Smad7 inhibition on TGF-β induced Smad1/5 phosphoylation and Smad2 phosphorylation in BAECs. BAECs were infected with adenoviral constructs of Flag-Smad7, or LacZ as a control, with MOI of 400. Forty hours after infection, BAECs were stimulated with 1 ng/ml of TGF-β for different time periods at 37°C before lysis. Whole cell lysate was sonicated and fractionated by SDS-PAGE and blotted. The filters were incubated with PS1, PS2 and Flag antibodies. (*C*) MEECs were co-transfected with caALK1 and BRE-luc with or without Smad7 at different concentrations. Luciferase activity was measured 48 h after transfection. Values are corrected for transfection efficiency as measured by β-galactosidase activity. A representative experiment using triplicate samples is shown. (*D*) Smad7-RNAi plasmid with a hygromycin cassette was stably transfected in MEECs and selected on hygromycin medium for 7 days. PGK-hygromycin selected cells were used as mock cells. The cells were serum-starved overnight and stimulated with TGF-β (1 ng/ml) at different time periods prior to lysis, sonication and fractionation by SDS-PAGE. Gels were then subjected to immunoblotting. The filters were incubated with PS1, PS2, or actin antibody. (*E*) *Left panel*. MEECs were transfected with BRE-luc in the absence or presence of Smad7-RNAi. Forty-eight hours after transfection the cells were washed extensively. Luciferase activity was measured after 8 hours of stimulation of TGF-β or not. Values are corrected for transfection efficiency as measured by β-galactosidase activity. A representative experiment using triplicate samples is shown. *Right panel*. MEECs were transfected with (CAGA)_12_-luc in the absence or presence of Smad7-RNAi. Forty-eight hours after transfection the cells were stimulated for 16 hours with TGF-β and luciferase activity measured. A representative experiment using triplicate samples, corrected for transfection efficiency, is shown.

The inhibitory Smad7 is known to be upregulated by TGF-β [8]. Our data imply that the negative regulation of TGF-β-induced Smad1/5 signaling needs newly synthesized protein to become effective and Smad7 may thus possibly mediate this effect. We therefore examined Smad7 expression levels in ECs by semi-quantitative RT-PCR upon infection of adenovirus expressing constitutively active (ca)ALK1, caALK5, or noninfected cells that were stimulated with TGF-β. Fig. 3A shows that Smad7 mRNA was upregulated upon stimulation of cells with caALK1 and TGF-β (0.25 ng/ml), and peaked at 30 min to 1 hour of TGF-β stimulation (data not shown). This bell-shaped dose-response curve is consistent with a previous observation that TGF-β-induced Smad1/5 phosphorylation is most prominent at 0.5–1 ng/ml with higher doses of TGF-β having less Smad1/5 activation [3]. Notably, caALK5 did not induce Smad7 expression.

### Smad7 is a highly efficient negative regulator of TGF-β/ALK1 signaling in ECs

Next, we investigated whether overexpression of Smad7 affects TGF-β/ALK1 signaling. The cells were infected with adenovirus expressing either lacZ or Flag-Smad7 at the MOI of 1000 and the effect on Smad phosphorylation was analysed after subjecting the cells to different concentrations of TGF-β. Smad7 overexpression blocked both Smad1/5 and Smad2 phosphorylation (Fig. 3B, *left panel*). Interestingly, if cells were infected with F-Smad7 at a MOI of 400, resulting in cells with lower levels of ectopically expressed Smad7 protein, the ALK1/Smad1/5 pathway but not the ALK5/Smad2 pathway was inhibited (Fig. 3B, *right panel*). We also examined whether Smad7 can inhibit Smad-mediated transcriptional responses in ECs. Smad7 inhibited TGF-β/ALK1-induced BRE-luc activity in a dose dependent manner (Fig. 3C). TGF-β-induced Smad1/5 phosphorylation was also examined when endogenous Smad7 expression was specifically inhibited. This was done by stable transfection in MEEC with siRNA-Smad7 plasmid including a hygromycin cassette. TGF-β-induced Smad1/5 phosphorylation was found to be prolonged in MEECs stably transfected with siRNA-Smad7 compared to control (PGK-hygromycin transfected) cells (Fig. 3D). Consistent with this finding, upon siRNA-mediated knockdown of Smad7, TGF-β-induced BRE-luc reporter activation was significantly enhanced. siRNA-mediated knockdown of Smad7 moderately inhibited TGF-β/ALK5 signaling using (CAGA)_12_-luc as read-out (Fig. 3E, *right panel*). The latter effect is likely indirectly caused by increased TGF-β/ALK1 signaling that antagonizes ALK5 signaling [4]. Taken together, these data indicate that Smad7 is enhanced by TGF-β/ALK1 signaling and that it is highly effective in inhibiting the same pathway.

### Inhibition of proteasome and protein phosphatase activity prolongs the TGF-β-induced Smad1/5 phosphorylation in ECs

To examine the involvement of the proteasome pathway in negative regulation of TGF-β/ALK1 signaling we treated BAECs with the proteasome inhibitor MG-132, and observed that Smad1/5 phosphorylation was stronger and prolonged compared to the non-treated cells (Fig. 1A, *third panel*). Interestingly, when we treated BAECs with the serine/threonine phosphatase inhibitor, calyculin, Smad1/5 phosphorylation was sustained (Fig. 1B). We also treated the BAECs with the phosphatase inhibitor, orthovanadate for 30 min. TGF-β-induced Smad1/5 phosphorylation was stronger and prolonged compared to non-treated cells (Fig. 1A, *fourth panel*). Orthovanadate is frequently used as a tyrosine phosphatase inhibitor, but certain PPs, such as the PP1 serine/threonine phosphatase, are also potently inhibited by orthovanadate [23]. These findings suggest that proteasome degradation and dephosphorylation by PPs play prominent roles in inhibiting the activation of TGF-β-induced Smad1/5 phosphorylation. As the involvement of proteasome pathway, but not PPs, had been intensely investigated in TGF-β signaling, we focused our subsequent studies on the involvement of PPs in TGF-β/ALK1 signaling.

### PP1α, which is transcriptionally induced by ALK1 activation, negatively regulates ALK1 signaling in ECs

**Figure 4:**
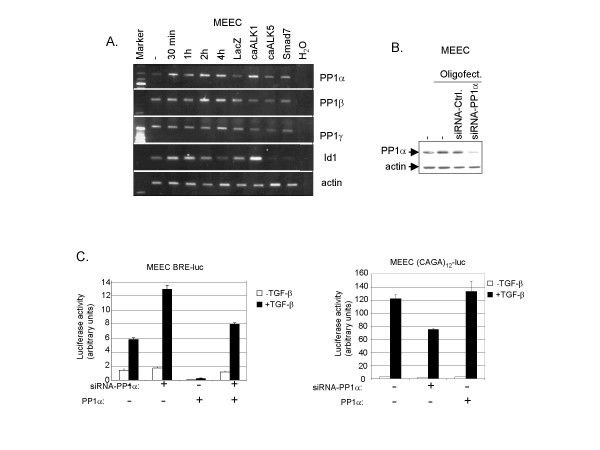
**ALK1-induced PP1α negatively regulates transcriptional activity downstream of TGF-β/ALK1.**(*A*) TGF-β kinetics and upregulation of PP1 isoforms on mRNA level. MEECs were stimulated with TGF-β or adenovirally infected with LacZ, caALK1, caALK5 or Smad7 at MOI of 500. Forty hours later, including starvation overnight, the cells were lysed, RNA was isolated and cDNA was prepared. PCR-amplified products for PP1α, β and γ and Id1 are indicated on the right of the figure. β-actin was included as a loading control. (*B*) MEECs were transfected with siRNA-PP1α using oligofectamine in medium without serum. Twelve hours later the transfection medium was changed to medium with 10% FCS. Forty-eight hours later, the cells were lysed. Whole cell lysate was sonicated, fractionated on SDS-PAGE and subjected to immunoblotting. The filter was incubated with antibodies against PP1α and actin to measure loading of proteins in each sample. (*C*) *Left panel*. MEECs were transfected with BRE-luc in the absence or presence of PP1α. Sixteen hours later the cells (where indicated) were transfected with siRNA-PP1α using oligofectamine. Forty-eight hours later, luciferase activity was measured 6 h after stimulation with TGF-β or not. Values are corrected for transfection efficiency as measured by β-galactosidase activity. A representative experiment using triplicate samples is shown. *Right panel*. MEECs were transfected with (CAGA)_12_-luc in the absence or presence of PP1α. After 16 h, the cells was transfected with siRNA-PP1α (where indicated) using oligofectamine. Thirty-two hours later, cells were incubated with TGF-β for an additional 16 h, whereafter luciferase activity was measured. Values are corrected for transfection efficiency as measured by β-galactosidase activity. A representative experiment using triplicate samples is shown.

Based upon our results implicating PPs in the negative regulation of TGF-β-induced ALK1/Smad1/5 phosphorylation in ECs and a previous report about the involvement of PP1 in dephosphorylation of the *dpp *type I receptor [20], we decided to look further into the connection of PP1 in TGF-β/ALK1 signaling. PP1 isoforms were expressed at the mRNA level in MEECs using specific sets of primers (data not shown, see Material and Methods). The effect of ALK1 or ALK5 activation on PP1 expression was investigated. Figure 4A demonstrates that only PP1α is strongly upregulated in MEECs expressing caALK1. PP1α mRNA was found to be upregulated upon caALK1 expression. Subsequently, we analysed the effect of TGF-β on P1α expression in time (Fig. 4A); PP1α was already upregulated after 30 minutes of TGF-β stimulation. Analysis of Id1, a direct target gene of ALK1 signaling, was taken along as a control. These data suggest that like Smad7, PP1α is a direct ALK1 target. However, analysis of the effect of TGF-β on PP1α protein expression did not reveal any upregulation (data not shown). The significance of PP1α in negative regulation of TGF-β-induced Smad1/5 activation was shown by siRNA-mediated knockdown studies of PP1α (Fig. 4B). TGF-β/ALK1-induced transcriptional activity downstream of the BRE-luc reporter was greatly enhanced upon specific inhibition of PP1α expression (Fig. 4C, *left panel*). Basal PP1 levels (and cooperating basal Smad7 levels, see below) are not sufficiently high for TGF-β/Smad1/5 signaling not to initially proceed. Conversely, transcriptional activity downstream of ALK5 was not affected by PP1α knockdown (Fig 4C, right panel). The latter is consistent with our finding that TGF-β/ALK5 -induced Smad2 phosphorylation (compared to Smad1/5 phosphorylation) is sustained in ECs.

### PP1α binds strongly to ALK1 in the presence of Smad7 and dephosphorylates the ALK1 kinase

**Figure 5:**
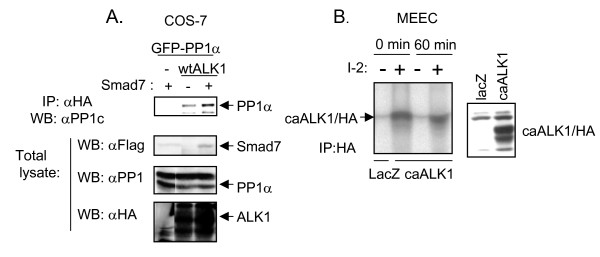
**Smad7 recruits PP1α to ALK1. **(A) COS-7 cells were transfected with eGFP-PP1α, wtALK1/HA and/or Flag-Smad7. After 48 h at 37°C, cells were lysed. Whole cell lysates were immunoprecipitatedwith HA antibody, fractionated by SDS-PAGE, and subjected to immunoblotting with PP1c antiserum. Total lysates were fractionated by SDS-PAGE and subjected to immunoblotting with antisera against Flag, PP1 and HA to check expression levels. (*B*) *Left panel*. Phosphatase inhibition assay. MEECs were adenovirally infected with the indicated constructs and immunopreciptated with HA antibody. Kinase assay was performed with 2.5 μCi of [^32^P]γ ATP per sample at 30°C for 30 min. Samples were washed in lysis buffer before they were incubated in a phosphatase buffer with or without the PP1 inhibitor, I-2 at 37°C for 60 min. The samples were separated by SDS-PAGE and phosphorylation quantified using phosphoimager. *Right panel*. Fraction of the total lysate was immunoblotted with HA antiserum to check expression levels of the receptor.

Subsequently, we examined whether PP1α could interact with ALK1. We co-expressed wild-type (wt) ALK1/HA and eGFP-PP1α with or without Flag-Smad7 in COS-7 cells. Cell lysates were subjected to immunoprecipitation for wtALK1/HA followed by immunoblotting for PP1 (Fig. 5A). PP1α was found to interact with ALK1, and Smad7 further enhanced this interaction. Consistent with these results, ^125^I-TGF-β cross-linked to ALK1 was co-immunoprecipitated with eGFP-PP1α when ALK1 and eGFP-PP1α were ectopically expressed in 293T cells (data not shown). Next, we analysed if endogenous PP1 affected the phosphorylation of ALK1. MEECs were adenovirally infected with caALK1/HA and the whole lysate was immunoprecipitated with HA antibodies. The samples were washed and subjected to an *in vitro *kinase assay followed by a phosphatase assay in the absence or presence of a specific PP1 inhibitor, Inhibitor-2 (I-2). As shown in Figure 5B, ALK1 was phosphorylated in the presence of I-2, whereas no ALK1 phosphorylation was evident in the absence of I-2. Taken together, these results indicate that PP1α interacts with and dephosphorylates ALK1.

## Discussion

In the present study we have investigated the molecular mechanisms that underlie the transient Smad1/5 activation by TGF-β/ALK1 signaling versus the sustained TGF-β/ALK5-induced Smad2 activation in ECs [3]. Our results support a model in which the inhibitory Smad7 is specifically induced by the TGF-β/ALK1 pathway and participates in a negative feedback loop in ECs. A rate equation model for the TGF-β/Smad pathway in ECs showed that Smad7 feedback loop provides robustness to the system [24]. Smad7, previously shown to be capable of interacting with type I receptors [6,8], may recruit PP1α to ALK1. The serine phosphatase activity of PP1α mediates dephosphorylation and inactivation of ALK1.

Duration of TGF-β/Smad signaling is a critical determinant for regulating specificity of cellular biological responses [2]. During *Xenopus *embryogenesis differences in the duration of Smad signaling is carefully controlled since it is important for cell fate decisions [25]. Whereas epithelial cells with a sustained Smad response are arrested in growth by TGF-β, pancreatic tumors that demonstrate a transient TGF-β/Smad response have specifically evaded anti-proliferative effects of TGF-β, while maintaining other TGF-β responses [26]. TGF-β/ALK1 signaling promotes the activation states of ECs. A transient versus sustained ALK1 response, as determined by Smad7 and PP1α expression levels, could be of critical importance for angiogenesis. After ECs receive a TGF-β/ALK1 signal and start to proliferate, migrate and form sprouts, this signal must be turned off, whereafter the TGF-β/ALK5 signal dominates and the maturation of vessels is induced. Interestingly, PP1 (and 2A) activity was previously shown to be needed to maintain ECs in a resting state [27]; inhibition of PP1c activity promoted EC migration consistent with a negative role for this phosphatase in TGF-β/ALK1-induced activation of ECs.

Here we show that in ECs Smad7 is more efficiently induced by ALK1 than by ALK5. In addition, Smad7 is more efficient in blocking signaling via ALK1 than ALK5. Previously, Smad7 was shown to be induced by, and to be a general inhibitor of, TGF-β superfamily signaling in various non-ECs [14,28,29]. Specific factors present in ECs are likely to be the reason why Smad7 is much more important feedback inhibitor downstream of ALK1 than ALK5 signaling in ECs, compared to TGF-β and BMP signaling in other cell types.

The proposed mechanism by which PP1 mediates the dephosphorylation of ALK1 in ECs is reminiscent to that recently reported for the recruitment of PP1 by Smad7 to ALK5 in epithelial cells [22]. Shi and co-workers reported that Smad7 interacts with GADD34, a regulatory subunit of PP1 holoenzyme. PP1c is recruited to ALK5 via a Smad7-GADD34 complex and then dephosphorylates activated ALK5. SARA enhances the recruitment of PP1c to the Smad7-GADD34 complex by enhancing the availability of PP1c to the Smad7-GADD34 complex. Which regulatory subunit of PP1α holoenzyme cooperates with Smad7 to interact with ALK1 remains to be investigated.

## Conclusion

Our results suggest that upon its induction by the TGF-β/ALK1 pathway, Smad7 recruits PP1α to ALK1, and thereby inhibit TGF-β/ALK1-induced Smad1/5 phosphorylation. Smad7 functions in different ways to exert its antagonistic effects. Besides the recruitment of PP1 by Smad7 to the phosphorylated type I receptor to dephosphorylate and inactivate it, Smad7 has been shown to compete with R-Smads for binding to activated type I receptors and thereby inhibit phosphorylation of R-Smads [6,8]. Our results do not exclude the possibility that ALK1/Smad signaling is subject to this type of inhibitory regulation by Smad7. In addition, Smad7 can recruit Smurf E3-ubiquitin ligases to the activated type I receptor, resulting in receptor ubiquitination and degradation [12,13]. Treatment of ECs with proteasome inhibitor revealed that TGF-β/ALK1 signaling is also negatively regulated by the proteasome pathway (Fig. 1); whether this occurs at receptor or Smad level remains to be elucidated. Further experiments are needed to examine the contribution of PP1 compared to other mechanisms of negative control by Smad7 in TGF-β/ALK1 signaling.

## Methods

### Ligands, antibodies and inhibitors

Recombinant TGF-β 3 was obtained from K. Iwata (OSI Pharmaceuticals). Phospho-Smad1/5 and phospho-Smad2 antibodies that specifically recognize phosphorylated Smad1/5 and phosphorylated Smad2 have been previously described [3,30], Smad5 rabbit antisera [31], Flag antibodies (SIGMA), HA antibodies (Roche), α-actin antibodies (Chemicon), goat-PP1α (Santa Cruz) and mouse-PP1c antibodies (Santa Cruz), were used in immunoblotting. Cells were pre-treated prior to stimulation with TGF-β and during the stimulation with cyclohexamide (SIGMA), MG-132 (Calbiochem), sodium orthovanadate (SIGMA-Aldrich) or calyculin (Calbiochem). In the phosphatase inhibition assay, a human recombinant protein phosphatase Inhibitor-2 (I-2) (Calbiochem), was added.

### Expression plasmids and RNAi

Constructs for TGF-β signaling components have been described previously (Goumans et al., 2002). Rabbit peGFP-C1 PP1α[32] was used in transient transfections. RNAi -Smad7 was made by cloning 5'-gatccccGACTCGCGTGGGGAGGCTCttcaagagaGAGCCTCCCCACGCGAGTCtttttggaaa-'3 and complementary oligonucleotides derived from mouse Smad7 in pSuper, RNAi-ALK1 was made by cloning 5' gatccccCACGGCTCCCTCTATGACTttcaagagaAGTCATAGAGGGAGCCGTGtttttggaaa-'3 and complementary oligonucleotides derived from mouse ALK1 in pSuper and RNAi-Smad5 was made by cloning 5'-gatccccGGTGTTCATCTATACTACGttcaagagaCCGTAGTATAGATGAACACCtttttggaaa-'3 and complementary oligonucleotides derived from mouse Smad5 in pSuper [33]. To silence endogenous PP1α expression, double-stranded 21-nt RNAs directed against Smad7 were chemically synthesized and purified (Qiagen). The siRNA-PP1α sequence was 5'-AAGACGUUCACUGACUGCUUC-'3.

### Cell culture

Bovine aortic ECs (BAEC) were routinely cultured in low-glucose DMEM (Gibco BRL) supplemented with 10% calf serum, L-glutamine and antibiotics. The cells were grown in a 10% CO_2_-containing atmosphere at 37°C. Mouse embryonic ECs (MEECs) were routinely cultured in DMEM supplemented with 10% fetal calf serum (FCS), non-essential amino acids, L-glutamine and penicillin/streptomycin on 0.1% gelatin-coated dishes. COS-7 and 293T cells were cultured in DMEM supplemented with 10% FCS, L-glutamine and penicillin/streptomycin. MEECs, COS-7 and 293T cells were grown in 5% CO_2_-containing atmosphere at 37°C.

### Transfections and transcriptional reporter assays

MEECs were transiently and stably transfected using lipofectamine™ reagent (Invitrogen) according to the manufacturer's protocol. COS-7 and 293T cells were transfected by conventional calcium phosphate co-precipitation method. In case of siRNA, the transfection was performed using oligofectamine™ reagent (Invitrogen) according to manufacturer's instructions. Reporter assays were performed as previously described [34]. MEECs were transfected with 0.5 μg BRE-luc [35] and 0.5 μg (CAGA)_12_-luc [36], in the absence or presence of an expression plasmid. In case of BRE-luc reporter, cells were stimulated with TGF-β for 6–8 h whereas (CAGA)_12_-luc transfected cells were stimulated for 16 h.

### Adenoviral infection of ECs

ECs were infected with adenoviruses expressing LacZ, wtALK1, caALK1, caALK5, Id1 and Smad7 at a multiplication of infection (MOI) of 500 unless else is indicated. After 16 h the cells were washed and allowed to recover for 8 h prior to starvation overnight before the indicated assays.

### Western blot analysis and immunoprecipitation

ECs were grown to 90% confluence. Cells were rinsed with PBS and grown in 0.5% FBS containing medium. After 16 h, cells were stimulated for 1 h with 10 ng/ml of TGF-β 3, put on ice, rinsed with PBS and lysed in lysis buffer (125 mM NaCl, 10 mM Tris-HCl, pH 7.5, 1 mM EDTA, 1 mM PMSF, 1.5% aprotinin and 1% Triton X-100). Cell lysates were separated by SDS-PAGE using 8% polyacrylamide gels, followed by wet-transfer of the proteins to Hybond-C extra nitrocellulose membranes (Amersham). Non-specific binding of proteins to the membrane was blocked in TBS-T (0.01 M Tris-HCl, pH 7.4, 0.15 M NaCl, 0.1% Tween-20) containing 3% dry milk. Primary antibodies were diluted 1000-fold in TBS-T and secondary horseradish peroxidase-conjugated goat anti-rabbit or mouse IgG antibody (Amersham) was used at a 10,000-fold dilution in TBS-T. Detection was performed by ECL. To detect heteromeric complex formation between Smad7 and PP1α, cell lysates from transfected COS-7 and MEECs were subjected to immunoprecipitation followed by Western blotting, as previously described [34].

### RNA isolation and reverse transcription polymerase chain reaction (RT-PCR)

Total RNA was isolated from MEECs using RNeasy columns (Qiagen) according to manufacturer's instructions. RT-PCR reactions were performed as described by Goumans *et al*. (3). The PCR reactions were performed using a PTC-200 Peltier thermal cycler (MJ Research). The DNA sequences of the PCR primers that were used are available on request.

### Phosphatase assay

Cells were infected with the indicated viruses. After lysis, immunoprecipitation was performed using anti-HA antibodies, followed by *in vitro *kinase assay as described by Itoh *et al*. [37] using kinase buffer (40 mM Hepes, pH 7.4, 40 mM MgCl_2_, 2 mM MnCl_2_, 2 mM DTT) including 2.5 μCi of [^32^P]γ ATP, and an incubation time of 30 min. Samples were washed with lysis buffer before they were subjected to phosphatase buffer (50 mM Tris, [pH 7.5], 1 mM EDTA, 0.1% β-mercaptoethanol, 1 mg/ml BSA) and incubated with or without Inhibitor-2 (Calbiochem) (10 ng/ml) at 37°C for 1 h. Protein samples were separated by SDS-PAGE using 8% polyacrylamide gels, followed by fixation and detection on a phosphoImager.

## Authors' contributions

GV performed the TGF-β-induced Smad phosphorylation assays, expression analysis of TGF-β signaling components and drafted the manuscript.

M-JG performed the transcriptional reporter assays.

FI and SI carried out immunoprecipitation assays.

C-HH helped design and evaluate biochemical assays.

PtD coordinated the experimental work and finalized the manuscript.

All authors read and approved the final manuscript.
